# Mineral acquisition from a different angle – how the root angle in cereals determines nutrient uptake

**DOI:** 10.1111/nph.70466

**Published:** 2025-08-11

**Authors:** Frederik J. T. van der Bom, Lorene Siegwart, Giuseppe Sangiorgi, Gwendolyn K. Kirschner

**Affiliations:** ^1^ Section of Crop Science, Department of Plant and Environmental Sciences University of Copenhagen Højbakkegaard Allé 13 DK‐2630 Taastrup Denmark; ^2^ Department of Agricultural and Food Sciences University of Bologna 40127 Bologna Italy; ^3^ The James Hutton Institute Invergowrie Dundee DD2 5DA UK

**Keywords:** nutrients, root angle, root ideotype, root plasticity, root system architecture, root traits, rooting depth, soil exploration

## Abstract

Plant roots are vital for absorbing soil resources and directly impact crop productivity. Because nutrients are unevenly distributed through soil layers, root system architecture (RSA) is a key trait influencing nutrient uptake. Shallow RSA can enhance access to surface nutrients, while steeper architectures help reach deeper nutrients. A central factor in RSA is root angle, which determines how roots spread through the soil profile. Although recent studies have investigated how the root angle affects nutrient acquisition in cereals, direct evidence remains limited. This review examines the molecular mechanisms regulating root angle and the relationship between root angle and nutrient uptake in different experimental setups. We highlight major knowledge gaps, including inconsistent data on nutrient uptake, limited insights into root plasticity, and unclear agronomic relevance of root angle. Our analysis suggests the assumed importance of root angle in cereal nutrient uptake may be overstated. To strengthen this area, we recommend interdisciplinary research combining genetics, molecular biology, physiology, and agronomy. Such efforts could lead to a more nuanced understanding and effective use of root angle traits in crop improvement strategies.

## I. Introduction

Agriculture needs to sustainably produce enough nutritious food without harming the environment. Due to climate change and unpredictable weather, we must shift to more resilient crops that simultaneously use fewer chemical fertilizers (Foley *et al*., 2011). Under these conditions, crop productivity will depend on how well the plants acquire water and nutrients from the soil. Plant roots play a crucial role in this process, serving as a key driver of soil nutrient uptake.

Root system architecture (RSA), that is the spatial configuration of roots, determines how roots are distributed along the soil profile and consequently, how plants access soil nutrients (Fitter, 1987). RSA is shaped by three key processes: formation of the different root types, root elongation, and root orientation in relation to gravity (Fitter, 1987). In cereals, the root system initially develops the embryonic primary root that grows at a vertical angle and seminal roots that emerge after germination and grow at shallower angles. Shoot‐borne roots develop from successive under‐ and aboveground nodes of the stem, known as adventitious roots (crown/nodal or brace roots), whose angles depend on their nodal position (York & Lynch, 2015). All root types then develop lateral roots (Hochholdinger *et al*., 2004). In tillering species, tillers emerge from the leaf axils at these nodes around the same time and can initiate their own root systems following a similar developmental pattern as that of the main stem, though with a relative delay (Klepper *et al*., 1984).

**Fig. 1:**
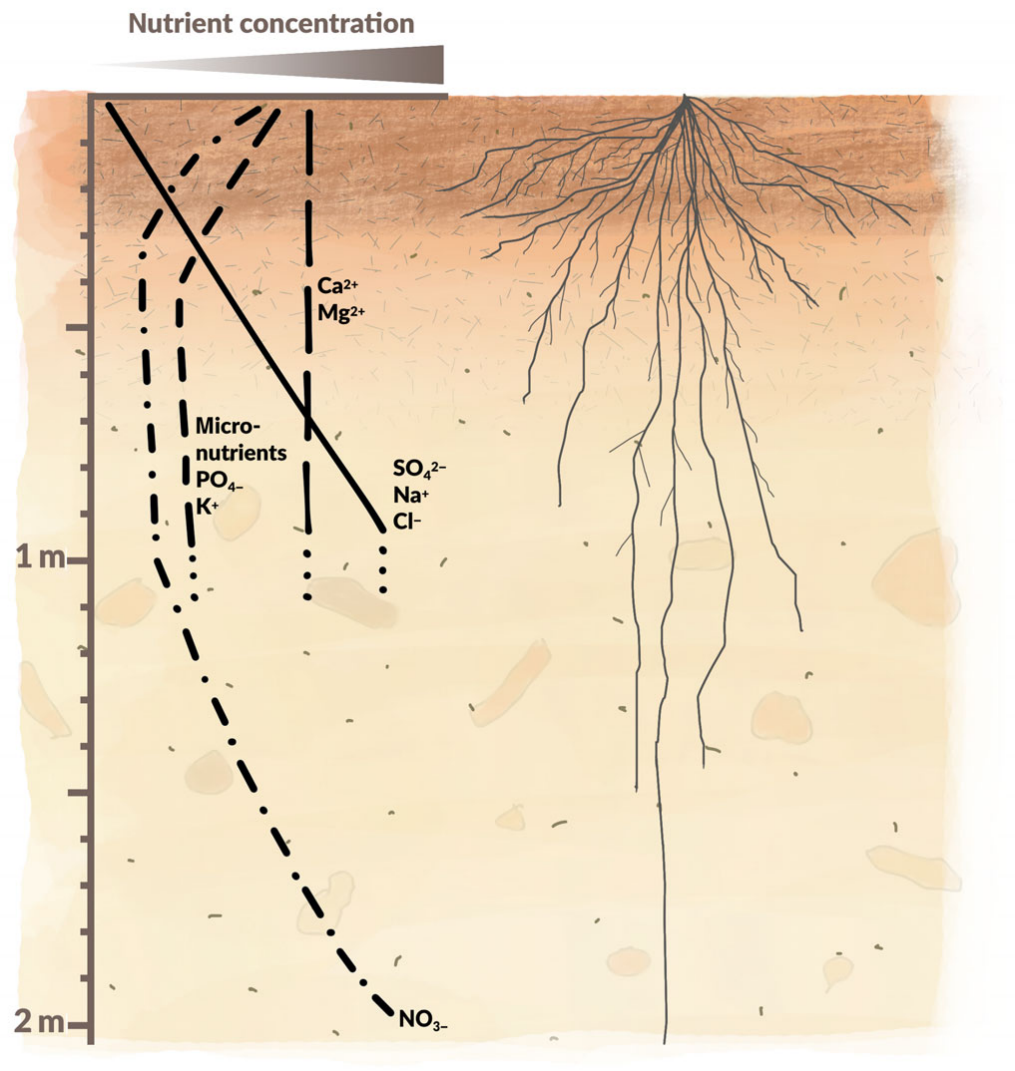
Minerals are unevenly distributed across the soil profile. Nutrient distributions and availability across the soil profile vary with several factors such as fertilizer application, plant uptake, bonding with soil particles, microbial activity, weathering, and leaching. Certain nutrients are more easily leached into the soil due to their chemical properties (solubility and electrical charge), which affect their relative mobility in soil. Furthermore, soil conditions such as the soil texture, the cation exchange capacity, and the amount of water (rainfall) are of major influence on the leaching process. In many soils, phosphorus (P), potassium (K), and micronutrients tend to be found in the topsoil, although K leaching may be substantial in sandy and organic soils in high rainfall areas. Cations such as calcium (Ca) and magnesium (Mg) tend to be evenly spread throughout the soil but can be susceptible to leaching. Sulfate (S), sodium (Na), and chloride (Cl) tend to be more concentrated in deeper layers. Nitrogen (N) is often applied and therefore mostly located at the soil surface; but being highly mobile in the form of nitrate (NO_3_^−^), it can leach into deeper layers as the growing season progresses (Angle *et al*., 1993; Franzluebbers, 1996; Jobbágy & Jackson, 2001; Dhaliwal *et al*., 2023). In addition, interactions can exist among different elements. For example, leaching of NO_3_ can result in accompanying leaching of Ca and Mg because of the need to maintain charge balance in the leachate. Similarly, large K applications can displace Ca and Mg from soil exchange sites, making them more susceptible to leaching. Cereal root systems often accumulate most of their root biomass at a depth of 0–20 cm but have been observed to reach 2.50 m into the ground (Fan *et al*., 2016; Siegwart *et al*., 2022).

It has been proposed that modifying RSA through breeding may deliver more nutrient‐efficient crops, especially under challenging conditions (Lynch, 2019). This involves selecting root traits that direct root growth toward soil layers in which scarce nutrients are more available. Among these traits, the root angle plays a central role by determining how roots grow in relation to gravity, influencing the effective depth and spatial distribution of the root system. Roots that grow at a steeper angle (more vertically) should penetrate deeper into the soil, enabling plant access to mobile elements that are prone to leach out of the root zone, such as nitrogen (N) and sulfur (S), and to a lesser degree potassium (K), calcium (Ca), and magnesium (Mg) depending on local conditions (Fig. 1). Conversely, roots that grow at a shallower angle (more horizontally) should spread out near the soil surface, enabling absorption of less mobile nutrients that tend to be more concentrated in the topsoil, such as phosphorus (P) (Fig. 1) (Oo *et al*., 2021).

Variation in root angle among cereal crops likely reflects adaptation to the diverse environments in which they were domesticated, including temperate and tropical climates. This diversity offers potential insights into how narrow or shallow root angles contribute to nutrient acquisition and under which conditions they may be most advantageous. Since root angle is genetically controlled, recent studies and (pre)breeding efforts have aimed to select crops with steep or shallow root angles (Maccaferri *et al*., 2016). However, apart from genetic control, plants can also modify their RSA depending on their environment (Grossman & Rice, 2012). This phenotypic plasticity means that the root angle can be adapted to different conditions – possibly within a genotypically determined range.

In this review, we examine the role of the root angle in nutrient uptake of cereal crops – and by extension, in addressing the challenge of sustainable and resilient crop systems. With an estimated global production of 2.85 billion tonnes in 2024 (*c*. 335 kg per person), cereals (including rice, wheat, rye, oats, barley, millet, maize, and sorghum) provide a key proportion of global dietary energy and nutrients demand (FAO, 2025). It is therefore vital to maintain and even boost their yields at minimal environmental impact. The review builds upon previous ones that discussed closely related issues, such as opportunities of root phenotypes for improved nutrient capture in agroecosystems (Lynch, 2019, 2022), root architectural trade‐offs for resource capture (van der Bom *et al*., 2020), root plasticity (Schneider & Lynch, 2020), genetic regulation of root angle in cereals (Kirschner *et al*., 2024), and prospects for breeding to improve root growth angle (Uga *et al*., 2015). We further acknowledge that relevant research has been conducted in other crops, particularly in common bean (Lynch, 2019 and references herein). Our review, however, focuses specifically on root angle in cereals and its role in determining the uptake of nutrients from soil. We first discuss the current assumptions and knowledge about molecular mechanisms that regulate root angle and about relationships between root angle and nutrient uptake in soil. We then identify knowledge gaps and finally discuss potential pathways to fill them.

## II. What do we know?

### 1. Molecular mechanisms that regulate the root angle

Understanding the molecular control of the root angle is fundamental for breeding crops with optimal root architecture. Root angle regulation occurs at the genetic level (Kirschner *et al*., 2024) and is determined by the root gravitropic set‐point angle – the angle in relation to gravity (Digby & Firn, 1995). The gravitropic set‐point angle is determined by the balance between gravitropic (toward gravity) and antigravitropic (against gravity) mechanisms (Fusi *et al*., 2022), which have mostly been studied in Arabidopsis lateral roots, but recent work has started to uncover mechanisms in cereal crops (Kirschner *et al*., 2024).

**Fig. 2:**
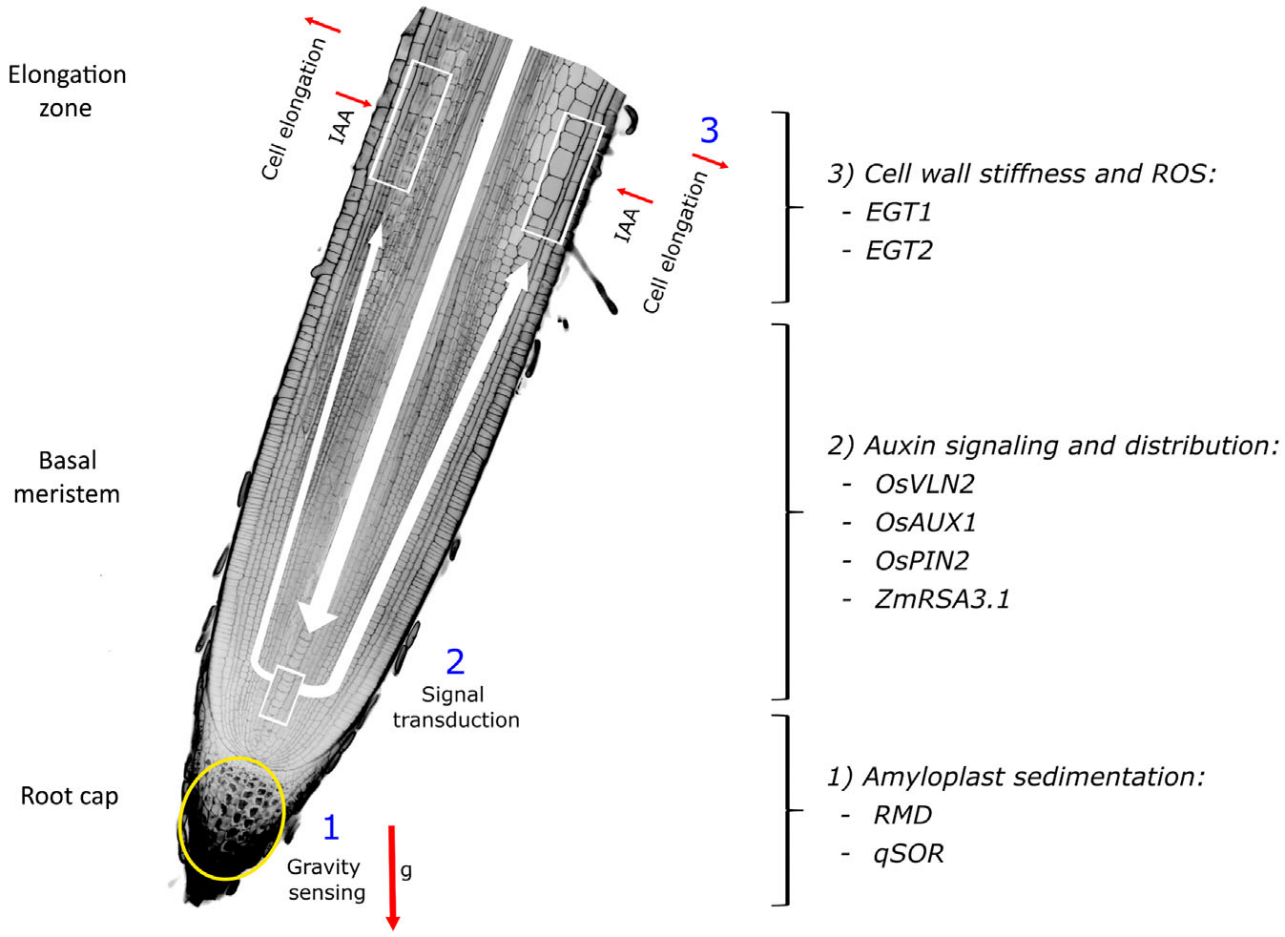
Root angle regulation is based on gravity response and occurs in the root tip. Cereal root tip with the key functional zones for gravitropism signaling: the root cap (yellow circle) is responsible for gravity sensing, triggering signal transduction in the basal meristem. Indole‐3‐acetic acid (IAA) moves upwards and is asymmetrically redistributed (white arrows) based on gravity direction. This lateral redistribution regulates cell elongation in the elongation zone. The red arrow indicates gravitational force (g). The genes listed on the right correspond to previously identified cereal genes and the molecular processes in which they are involved.

Gravity response can be divided into gravity sensing, signal transduction, and differential cell elongation (Su *et al*., 2017). Gravity sensing occurs at the root tip, mediated by starch‐filled amyloplasts (statoliths), which sediment at the bottom of columella cells in response to gravity (Huang *et al*., 2018), leading to asymmetric auxin distribution. Auxin accumulation at the lower side of the root then induces differential cell elongation in the elongation zone, causing the root to grow downward (Fig. 2) (Su *et al*., 2017). Several genes are involved in those pathways in cereals, disruptions of which can alter the gravity response and thereby the root angle.

Researchers have identified various genes in cereals that are involved in sensing gravity. For example, the actin‐binding protein Rice Morphology Determinant (RMD) is involved in buffering the amyloplast sedimentation in rice (Huang *et al*., 2018). In *rmd* mutants, amyloplasts sediment faster than in the wild‐type (WT), causing a steeper root angle. LAZY1‐LIKE proteins transmit the statolith position information to the plasma membrane, creating polarity and triggering asymmetric auxin distribution through interactions with regulator of chromosome condensation1‐like domain proteins and the auxin efflux carriers PINFORMED3 (PIN3) (Nishimura *et al*., 2023). The *LAZY* homolog genes, *DEEPER ROOTING 1* (*DRO1*) and *quantitative trait locus for SOIL SURFACE ROOTING 1* (*qSOR1*), regulate the root angle in rice, putatively by manipulating gravity sensing (Uga *et al*., 2013; Kitomi *et al*., 2020). Combinations of gain‐of‐function and loss‐of‐function alleles in *qSOR1* and *DRO1* cause different root angle phenotypes, ranging from shallow to steep.

Signal transduction from the site of gravity sensing to the elongation zone involves the formation of a lateral auxin gradient and calcium transients (Su *et al*., 2017). In rice and maize, several proteins contribute to this process, including auxin carriers and cytoskeleton‐related proteins. For instance, mutants of auxin carriers exhibit shallower root angles due to compromised gravitropic responses (Wang *et al*., 2018; Giri *et al.*, 2018). Additionally, mutations in the gene encoding the actin‐binding protein VILLIN2 (OsVLN2) disrupt OsPIN2 recycling, which affects auxin distribution and results in an exaggerated gravitropic response (Wu *et al*., 2015). The maize gene *ZmRSA3.2* regulates actin and microtubule dynamics. Its overexpression enhances gravitropic responses by influencing auxin transport and actin rearrangement (Ren *et al*., 2022). Moreover, calcineurin B‐like protein (CBL)‐interacting serine/threonine‐protein kinase 15 (ZmCIPK15) may also be involved in calcium‐mediated gravity signal transduction interacting with calcium‐binding proteins (Schneider *et al*., 2022).

In addition to genes that regulate the gravitropic response, recent studies have identified genes involved in the antigravitropic response in barley and wheat. *ENHANCED GRAVITROPISM1* (*EGT1*), an F‐box protein with a Tubby domain, modulates root angle by affecting cell wall stiffness, counteracting gravitropic bending (Fusi *et al*., 2022). *EGT2* regulates the root growth angle by modulating the expression of genes related to cell wall structure and reactive oxygen species in the root tip (Kirschner *et al*., 2021; Guo *et al*., 2023). Functional knockout of the *EGT1* and *EGT2* genes – abolishing the antigravitropic response – leads to hypergravitropic root growth, that is a steep root angle and a faster gravitropic response (Kirschner *et al*., 2021; Fusi *et al*., 2022).

Many genes that play a role in gravity response and root angle regulation show a conserved function between cereal plant species, and even dicots. Examples are *EGT1* and *EGT2*, whose function is conserved between wheat, barley, and Arabidopsis (Kirschner *et al*., 2021; Fusi *et al*., 2022), and the *LAZY‐like* genes that are conserved between Arabidopsis and rice (Uga *et al*., 2013; Nishimura *et al*., 2023). Some genes, such as *EGT1* and *EGT2*, play a general function across all root types – barley mutants show a steeper angle in seminal, crown, and lateral roots (Kirschner *et al*., 2021; Fusi *et al*., 2022). By contrast, other genes act more specifically; for example, Z*mCIPK15* affects root angle only at certain nodal positions in maize mutants (Schneider *et al*., 2022).

Discovering more genes involved in the gravitropic and antigravitropic responses will facilitate the isolation of haplotypes or the creation of mutants and near‐isogenic lines (NILs) with a range of different root angles that can be used for experimental studies evaluating the role of the root angle for nutrient uptake. An example of this is the NIL of *DRO1* in rice, which exhibits a steeper root angle than the lines carrying the WT allele and is used for analyzing phosphate uptake and drought tolerance (Uga *et al*., 2013; Oo *et al*., 2021).

### 2. Relationships between root angle and nutrient uptake

Initial research on root angle in cereals explored correlations with various measures for vertical root distribution, such as the ‘root depth index’ (Oyanagi *et al*., 1993), ‘deep root ratio’ (Kato *et al*., 2006), or the ‘fractal dimension’ (Manschadi *et al*., 2008). Related studies observed that drought‐adapted genotypes tended to possess steeper roots (Manschadi *et al*., 2008; Mace *et al*., 2012) and that steeper roots enhanced deep‐water uptake in simulations (Manschadi *et al*., 2006; Hammer *et al*., 2009). These combined insights support the hypothesis that a steep root angle may enhance the extraction of water – and by extension, the uptake of leached elements deeper in the soil profile. However, direct quantitative evidence remains limited (Table 1).

**Table 1:** Selected evidence for the role of root angle for rooting depth and water and nutrient uptake by cereals (in chronological order).

Study	Crop	Plant materials	Growth system(s)	Specific growth conditions	Duration	Key results	Root angle measured?
Oyanagi *et al*. (1993)	Wheat	12 cultivars from two geographic regions	Soil in pots/field		7 d/stem elongation stage	Correlation between root angle of seminal roots in pots and ‘root depth index’ (30 cm) in the field.	Seminal root angle. Basket method (pots)/soil monolith method (field)
Kato *et al*. (2006)	Rice	12 cultivars with contrasting root systems	Exp 1: field (2 yr) Exp 2: soil in pots, field (2 yr)	Exp 1: water stressed and irrigated y1/Exp 2: well‐watered	Exp 1: 110 d (y1), heading (y2)/Exp 2: 36, 60 d (y1), 53 d (y2)	Genotypic variation in nodal root diameter and root growth angle; association between ranked ‘deep root ratio’ and root angle.	Exp 1: no Exp 2: nodal root angle, basket method (pot and field)
Manschadi *et al*. (2008)	Wheat	30 cultivars from diverse regions	Exp 1: gel‐filled chambers; Exp 2: rhizoboxes	Unwatered	8 d/40 d	Variation in growth angle and fractal dimension (root distribution); most drought‐adapted cultivars clustered as steep root systems	Seminal root angle. For gel chambers only
Hammer *et al*. (2009)	Maize	Pioneer maize hybrid 3394	*In silico* (APSIM)	Locations across US corn belt	70‐yr simulations	Root angle and water capture related to biomass and historical yield trends	Parameterized
Mace *et al*. (2012)	Sorghum	Exp. 1: 141 RILs; Exp. 2: 44 diverse inbred lines	Soil in rhizoboxes		6th leaf stage	QTL root angle co‐locates with drought adaptation	Nodal root angle
Uga *et al*. (2013)	Rice	Shallow vs deep‐rooting NIL	Field	3 water levels	Maturity	Steeper root angle promoted deeper rooting and improved water uptake	Root depth by soil monolith sampling
Trachsel *et al*. (2013)	Maize	RILs (‘shallow’ vs ‘steep’ root angles)	Field	Low/high N, 2 locations	43 d, flowering, maturity	Root angle correlated with rooting depth (D95); N availability regulated root angle; no yield effect of root angle under Low N	Group means only (shovelomics)
Arai‐Sanoh *et al*. (2014)	Rice	Shallow vs deep‐rooting NIL	Field (2y)	Fertilizer yes/no (NPK)	20 d after heading (root coring), maturity (yield)	NIL greater root length at depth (20 cm), N uptake, and yield	No, refers to Uga *et al*. (2013)
Ali *et al*. (2015)	Maize	18 hybrids selected from a panel of 98	Exp 1: field; Exp 2 and 3: pots in a glasshouse	Exp1: two water levels	Exp 1: maturity/Exp 2: 10 d (1 leaf stage)/Exp 3: 33 d (6th leaf stage)	Phenotypic variations for seminal and nodal root angles; higher yielding hybrids under drought had steeper root growth angle than lower yielding hybrids; strong correlation between seminal and nodal root angles	Exp 1: no Exp 2: seminal root angle Exp 3: nodal root angle
York & Lynch (2015)	Maize	RILs with contrasting nodal root growth angles	Field	Low/high N, 2 locations, irrigated	72–73 d after planting	Root angle variable among node positions	Nodal root angle per whorl
Dathe *et al*. (2016)	Maize	Contrasting axial root growth angles	*In silico* (SimRoot)	7 N levels, 2 soils, 5 rain levels	42 d	Root angle influenced plant N capture; ‘optimal’ angle environment dependent	Parameterized
Robinson *et al*. (2018)	Barley	216 lines from breeding panel	Clear pot method; field		5 d; maturity	Both positive and negative correlation with yields, depending on environment.	Seminal root angle; subset of clear pots only
Alahmad *et al*. (2019)	Durum wheat	14 genotypes including NAM parents and bread wheat standards	Clear pot method; field		5 d; anthesis	Strong correlation between seminal root angle and mature root angle	Seminal root angle (pots); nodal root angle (field)
Bai *et al*. (2019)	Wheat	637 elite and breeding lines; subset of 72 lines	Cigar roll (637); wax layer (72); field (71)	2 field locations/yr	11 d/6 wk/heading	No relationship between laboratory‐measured root angle and field rooting depth	Distribution of seminal root angle in cigar roll screen only
Rich *et al*. (2020)	Wheat	20 genotypes with different emergence angles	Paper pouches; gel‐filled chambers; soil in pots; field		7 d/8 d/42 d/4 wk after flowering	Root angles from soil baskets correlated with rooting depth in the field; correlations inconsistent across methods and season	Yes
Oo *et al*. (2021)	Rice	NILs and their parents with shallow, steep, and intermediate root growth angles	Soil in rhizoboxes	P‐dipping (creating P hotspot at soil surface)	31 and 52 d after germination	Shallow root system improves applied P‐use efficiency	Yes
Schneider *et al*., (2022)	Maize	Mutant with steeper root angle than wild‐type	Medium filled mesocosms; field; *in silico* (opensimroot)	High/low N, High/low water	43–45 d/Flowering/40 d	Different angles in field and glasshouse; steeper angle improved biomass and N uptake under low‐N conditions (field and model)	Crown root growth angle
Liu *et al*. (2022)	Spring wheat	9 cultivars with diverse RSA traits	Field (2 yr)	Compaction yes/no	Stem elongation/flowering/maturity	Large year‐to‐year variation in root angle: steeper in dry year; greater N uptake efficiency and grain N concentration with shallow root angle	Root crown angle at stem elongation
Reyes‐Cabrera *et al*. (2023)	Sorghum	130 RILs	2 field sites x 2 yr	Exp 1: 4 N levels Exp: irrigation yes/no	Maturity	Minimal or no impact of RSA on N uptake and grain yields	No (refers to previously measured group means of crown root angle)
van der Bom *et al*. (2023a)	Durum wheat	2 contrasting genotypes ‘steep’ vs ‘shallow’ root angle	Soil in clear pot, rhizobox, and large mesocosm	Various P placements	5 d, late‐tillering, flowering	P dependent root angle plasticity Effect of root angle P and water dependent Root angle development stage dependent	Seminal root angle at three growth stages
van der Bom *et al*. (2023b)	Durum wheat, sorghum	Contrasting genotypes with steep vs shallow root angles	Soil in rhizobox	Various P placements	39 d (durum), 31 d sorghum	Effect of root angle dependent on P placement	Seminal root angle (durum) Nodal root angle (sorghum)
Kang *et al*. (2024)	Durum wheat	11 genotypes with steep vs shallow root angles	Medium in rhizobox		6 wk (late‐tillering stage)	Difference in root dry mass in the uppermost 30 cm of the soil: root angle correlated with root distribution	No, refers to Alahmad *et al*. (2019) and Richard *et al*. (2015) for information on seminal root angle
Oo *et al*. (2024)	Rice	2 distinct NILs with a shallow vs steep angle	Soil‐filled rhizoboxes	2 experiments with various P placements	21, 38, 42 d after transplanting	Deep‐rooted genotype greater root surface area in subsoil and greater biomass and P uptake under uniform high P; shallow‐rooted genotype: greater root surface area in topsoil and greater biomass and P uptake under uniform low P No genotypic differences for topsoil P conditions	Root growth angle in both experiments

NAM, nested association mapping; NIL, near‐isogenic line; QTL, quantitative trait locus; RIL, recombinant inbred line.

The relationship between a steep root angle and uptake of elements from deep soil layers and crop productivity is complex and varies among crops and environmental conditions. To date, studies have primarily focused on N, which is highly mobile in the form of nitrate. A steep root angle has been observed to have a positive effect on N uptake and biomass under low‐N but not high‐N conditions in maize (Schneider *et al*., 2022), a consistent positive effect on rice grain yield irrespective of low‐ or high‐N conditions (Arai‐Sanoh *et al*., 2014), or no effect under low‐N condition in maize (Trachsel *et al*., 2013). These results highlight a context dependency for potential benefits of a steep root angle, aligning with simulations that indicate a role for root angle in colocating roots and N, but that the ‘optimal’ angle depends on environmental conditions (e.g. soil type or precipitation; Dathe *et al*., 2016). Indeed, in some environments, a shallow rather than a steep root angle may enhance N uptake (Liu *et al*., 2022).

It is generally presumed that a shallow root angle should improve the uptake of nutrients with lower mobility, considering many cropping systems tend to exhibit greater availability of such nutrients in the topsoil (Jobbágy & Jackson, 2001). This stratification can be the result of shallow fertilizer applications and/or crop returns through litter, stubble, and throughfall. To date, studies investigating shallow nutrient uptake have mostly focused on P. Although these suggest that a shallow root angle may increase root surface area in the topsoil, the relationship with P uptake and crop biomass has varied with crop, soil P availability, and soil P distribution (Oo *et al*., 2021, 2024; van der Bom *et al*., 2023a,b). Specifically, the benefits of a shallow root angle under low‐P conditions and topsoil stratification have been inconsistent among studies, possibly due to differences in topsoil P availability. For example, severe P limitation may restrict plant root growth to such a degree that the amount of additional root surface area in the topsoil becomes negligible, or stratified P levels may be so high that root angle becomes less critical (van der Bom *et al*., 2023a,b). Additionally, placement depth may be important in low‐P environments with banded fertilizers, in which the optimal root angle may shift from shallow (Oo *et al*., 2021) to steep (van der Bom *et al*., 2023a,b) depending on the depth of a fertilizer band.

Thus, the relationships between root angle and nutrient uptake vary among crops and environmental conditions. Although there are promising insights, the variability highlights the need to fully understand the complex interactions to determine the optimal root angle for nutrient uptake for any given environment.

## III. What is missing?

### 1. Consistent data on root angle and its effects on nutrient uptake across crop growth and development

Despite extensive research, the evaluation of root angle, RSA, and spatial nutrient uptake remains incomplete. First, studies have primarily examined N as a mobile nutrient and P as a less mobile nutrient, broadly representing two distinct groups with contrasting behaviors and associated uptake strategies. Some of these results may be extended to other nutrients; however, caution is warranted because there can be substantial variation in mobility among elements and environments. For example, K is generally considered to be less mobile, but significant amounts may leach in light‐textured soils, such that subsoil K availability may contribute substantially to crop uptake (Hinsinger *et al*., 2021). Extrapolations between (groups of) elements therefore still require verification, especially considering evidence for a strong context dependency of root angle and its role on nutrient uptake.

Second, there is still a lack of studies that effectively connect laboratory phenotyping with field environments. Various laboratory approaches such as paper pouches, gel chambers, and rhizoboxes have offered easy access to roots, rapid and cost‐effective phenotyping and breeding for root angle (Richard *et al*., 2015), and the ability to relate root angle to the RSA of mature plants (Alahmad *et al*., 2019; Bai *et al*., 2019). However, many studies have short durations (Table 1), limited by small pots that can restrict root space and lead to potential artifacts (Poorter *et al*., 2012). Inconsistencies in root trait measurements are common among methods and plants of different ages, bringing a risk of overinterpretation when data are extrapolated to field conditions. For example, the root angle of young plants in pots could serve as an estimate for vertical distribution or even the root angle of the mature field root systems in some studies (Oyanagi *et al*., 1993; Kato *et al*., 2006; Ali *et al*., 2015), but others have been less conclusive (Watt *et al*., 2013; Bai *et al*., 2019; Rich *et al*., 2020; Schneider *et al*., 2022). Thus, it is still critical to validate laboratory findings and systematically quantify the implications of root angle across crop development.

Field trials play a critical role in understanding the impact of root angle on root growth, RSA, and nutrient uptake as root systems mature in various crop environments. However, studies face significant challenges such as high costs, difficulty of root sampling or accessing the root zone, and limited availability of high‐quality plant materials (e.g. mutants and NILs). These challenges often restrict trials to fewer genotypes or varieties, and fewer or simpler root measurements (e.g. Trachsel *et al*., 2011). Furthermore, the relationships between root angle and nutrient uptake or yield levels are highly variable (Robinson *et al*., 2018), likely influenced by environmental factors such as temperature, water, and nutrient availability. This variability highlights the importance of studying how environmental factors interact with root angle to impact nutrient uptake. The use of specialized semifield infrastructure (Svane *et al*., 2019; Thorup‐Kristensen *et al*., 2020) may offer a solution to this challenge, although such approaches may be out of reach for many institutions and regions with limited resources. Thus, there is still a pressing need for efficient, scalable, and cost‐effective alternatives that can be adapted to diverse contexts, to quantify these interactions and the role of root angle in nutrient uptake over time and space.

### 2. Understanding of root plasticity

Plants can dramatically adjust their RSA depending on environmental conditions such as soil compaction and the distribution of nutrients in soil. For example, P‐rich patches can elicit strong lateral root proliferation (van der Bom *et al*., 2023b), and N availability can significantly impact root angle (Trachsel *et al*., 2013). These responses may have major implications for the functioning of genotypes with selected root angles, considering soil nutrient heterogeneity is widespread in agricultural systems due to fertilizer application, cultivation, and soil biochemical processes. Additionally, studies indicate that plants can also modify their RSA in response to other plants. For instance, maize root angle can become steeper under high‐density crop stands (Shao *et al*., 2018). However, the underlying plant (molecular and physiological) and soil (biochemical) mechanisms and the broader implication for RSA and nutrient uptake remain poorly understood.

Root plasticity is potentially a heritable trait. One example is the regulation of the actin‐binding protein RMD, whose transcript expression is modulated by nutrient levels. Low P increases *RMD* expression, which reduces the asymmetry in statolith and auxin accumulation, resulting in a shallower root angle (Huang *et al*., 2018). However, selection under relatively homogenous, high‐fertility soil conditions may have reduced genetic variation, such that modern cereal crops may have a diminished capacity for root plasticity compared with their wild counterparts (Grossman & Rice, 2012). Therefore, it is crucial to include wild lines and landraces in studies and (pre)breeding programs to fully unravel the underlying molecular mechanism, which may be a starting point for exploiting root angle plasticity and adapting crops to agricultural practices.

Several studies have focused on the impact of environmental factors on root angle at the phenotypic level (Trachsel *et al*., 2013; van der Bom *et al*., 2023b). However, these studies often focus on single environmental factors and sometimes recommend genotypes with a particular root angle to improve uptake of a specific soil resource (Schneider *et al*., 2022). This approach may have limited consequences in systems in which other constraints have been mitigated through management, such as high‐input fertilizer applications. In this context, it has been argued that root plasticity should be selected against (Lynch, 2019). However, it remains unclear whether plasticity confers a benefit or imposes a cost in highly heterogeneous and unpredictable soil environments (Schneider & Lynch, 2020; van der Bom *et al*., 2020). While some studies link plasticity to improved uptake of limiting nutrients, our understanding is still limited regarding how spatiotemporal trade‐offs and feedback mechanisms influence RSA expression and nutrient acquisition across different soil regions (van der Bom *et al*., 2023a).

Given that roots are immobile once developed, root angle plasticity must be viewed not only in response to environmental cues at a single time point but also across developmental stages. Once a root segment grows at a particular angle, it remains fixed, and only the growing apex can adjust its trajectory. Therefore, assessments of root plasticity should include measurements at various points along the root to capture developmental changes. Focusing solely on the root angle at the point of emergence from the embryo or stem overlooks later‐stage adaptations. However, capturing these deeper root traits presents practical challenges, as roots may shift or distort during excavation, complicating accurate measurement.

### 3. Understanding the agronomic context

Ultimately, the productivity of genotypes with a selected root angle will depend on complex interactions between the crop, environment, and management in local conditions; extreme root architectures may only be beneficial under very specific environmental conditions (Dathe *et al*., 2016; van der Bom *et al*., 2020). The agronomic challenge is thus to determine the best conditions for any given root system and, potentially, to explore new management options to maximize the benefits of new varieties with selected root angles. Testing these questions would require further exploration of how root angles respond to varying environments, considering both the responses of individual root systems and those of entire crop stands or plant populations. In this context, crop systems such as cultivar mixtures or intercropping present a largely unexplored case, in which interactions between different root systems can be complex but potentially enhance nutrient uptake and crop performance (Hinsinger *et al*., 2011).

## IV. How do we get there?

**Fig. 3:**
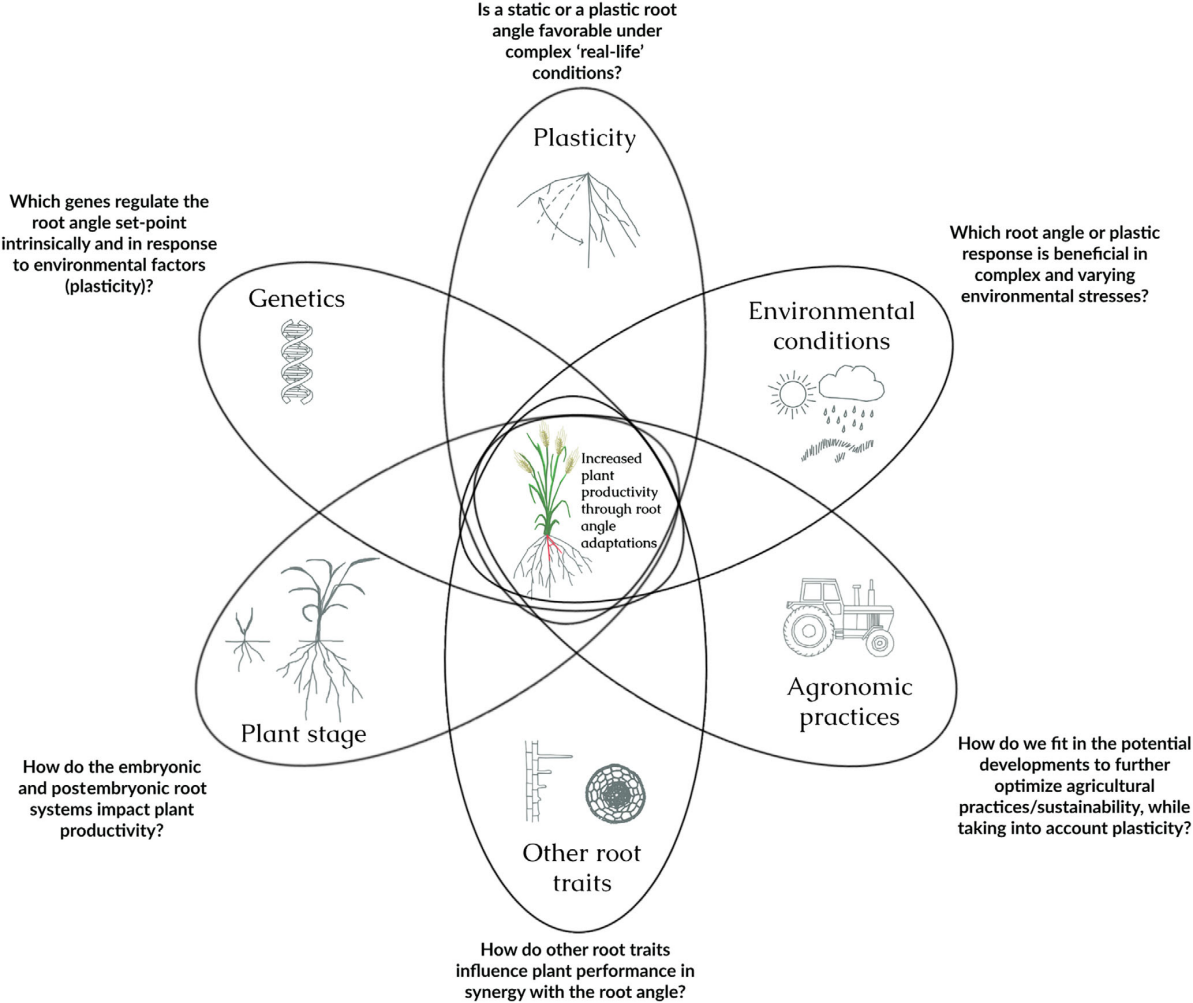
Understanding the root angle and its impact on plant performance requires the integration of different perspectives and expertise from different fields.

Despite ongoing advances in each discipline, the current lack of clear success stories underscores the need for more integrative research. Understanding root angle requires a multidisciplinary approach that combines molecular genetics, plant biology, soil science, and agronomy (Fig. 3). Extensive field trials are crucial to validate laboratory findings and evaluate the real‐world implications of root angle variation in agriculture. At the same time, controlled laboratory and glasshouse studies are essential for uncovering the mechanisms behind field observations. This integrated strategy will clarify the biological basis of root angle, reveal its effects on plant development and crop productivity, and enable the translation of insights into practical agricultural solutions.

We need to expand our fundamental understanding of the genetic and molecular processes that govern root angle regulation, to develop germplasm with diverse root angles. Forward genetics approaches including exploiting mutant collections and genome‐wide association studies with mild root angle variations can discover key genes involved in root angle regulation toward steeper or shallower root systems. This will enable the development of NILs with a wide range of root angles to test hypotheses and verify models independently of other genotype effects. In addition, it is important to understand how environmental factors such as the distribution of nutrients in soil will affect the root angle and thereby root distribution. For that, it is crucial to understand which genes regulate root angle plasticity, for example genes that are involved in sensing environmental cues or those involved in translating the cues to changes in root angle. This may be achieved by screening natural collections or mutant collections for root angle plasticity (i.e. which genotypes can better/less adapt angles to different conditions) and identifying the underlying genes. Furthermore, it is important to analyze root angles across plant stages to understand whether novel lines reliably express their root phenotype ‘as planned’ in varying environmental conditions, or whether creation of lines with a plastic root angle may deliver benefits by adapting to different unpredictable (varying and extreme) conditions.

*In silico* crop simulation models will need further development to incorporate root growth dynamics in response to environmental conditions. This will require extensive data on root behavior under various biotic and abiotic factors, that is the collection of datasets to accurately simulate these dynamics and improve model predictions. Precise identification of nutrient uptake, in space and time, would involve the use of isotopic tracers (e.g. ^15^N, ^32/33^P), while the impact of soil properties (e.g. structure, compaction, biopores, and nutrient heterogeneity) on RSA may be imaged *in situ* with magnetic resonance imaging or X‐ray computed tomography. Tracing spatiotemporal dynamics will unravel the importance of root growth and activity as the crop advances through different stages of phenological development, for example the colocalization of roots to access leached nutrients at later growing stages (Wacker *et al*., 2022). Enhanced simulation models would enable analyses of complex interactions and facilitate hypothesis testing regarding root angle and its effect on nutrient uptake and crop performance. For instance, the effects of seasonal climatic variability such as rainfall during crop growth and its synchrony with crop phenology are largely missing in the literature. Given the vast number of possible scenarios, simulations provide an indispensable alternative to extensive field experiments. Then, NILs with contrasting root angles could be used to test the hypotheses.

Apart from relationships between root angle and single factors, experimental setups should consider multiple stresses and growth conditions guided by agronomical practices. The combination of environmental conditions and agronomical practices can lead to complex trade‐offs and interactions. Developing an understanding of these interactions may be achieved by integrating glasshouse (e.g. pots or rhizotrons) field, and simulation experiments, with such studies further taking into account potential interactive effects of plant age and development stage. In addition, root angle determines root architecture in conjunction with many other traits such as root length, branching, diameter, tissue composition, and root type. These traits and other factors determining root–soil interaction (e.g. the microbiome) play important roles for access to soil nutrients. Integrating them together with aboveground traits and crop nutrient demand should help future decision‐making and potentially assist with the development of ‘tailor‐made’ root ideotypes to optimize nutrient capture and agricultural practices.

Ultimately, a comprehensive and integrative research strategy – spanning genetics to agronomy and simulations to field trials – is essential to fully elucidate the role of root angle in crop performance and to harness this trait for resilient, resource‐efficient agriculture.

## Competing interests

None declared.

## Disclaimer

The New Phytologist Foundation remains neutral with regard to jurisdictional claims in maps and in any institutional affiliations.
